# Increased Prefrontal Activation During Verbal Fluency Task After Repetitive Transcranial Magnetic Stimulation Treatment in Depression: A Functional Near-Infrared Spectroscopy Study

**DOI:** 10.3389/fpsyt.2022.876136

**Published:** 2022-04-04

**Authors:** Jiaxi Huang, Jiaqi Zhang, Tingyu Zhang, Pu Wang, Zhong Zheng

**Affiliations:** ^1^Mental Health Center, West China Hospital, West China School of Medicine, Sichuan University, Chengdu, China; ^2^Department of Rehabilitation Sciences, The Hong Kong Polytechnic University, Kowloon, Hong Kong SAR, China; ^3^Department of Rehabilitation Medicine, The Seventh Affiliated Hospital, Sun Yat-sen University, Shenzhen, China; ^4^Guangdong Engineering and Technology Research Center for Rehabilitation Medicine and Translation, Guangzhou, China

**Keywords:** major depressive disorder, repetitive transcranial magnetic stimulation, prefrontal cortex, functional near-infrared spectroscopy, verbal fluency task

## Abstract

**Background:**

Previous studies have shown the clinical effect of 2 Hz repetitive transcranial magnetic stimulation (rTMS) for depression; however, its underlying neural mechanisms are poorly understood. The aim of this study was to examine the effects of rTMS on the activity of the prefrontal cortex in patients with depression, using functional near-infrared spectroscopy (fNIRS).

**Methods:**

Forty patients with major depressive disorder (MDD) and 40 healthy controls were enrolled in this study. Patients underwent 4 weeks of 2 Hz TMS delivered to the right dorsolateral prefrontal cortex (DLPFC). fNIRS was used to measure the changes in the concentration of oxygenated hemoglobin ([oxy-Hb]) in the prefrontal cortex during a verbal fluency task (VFT) in depressed patients before and after rTMS treatment. The severity of depression was assessed using the Hamilton Rating Scale for Depression-24 item (HAMD-24).

**Results:**

Prior to rTMS, depressed patients exhibited significantly smaller [oxy-Hb] values in the bilateral prefrontal cortex during the VFT compared with the healthy controls. After 4 weeks of 2 Hz right DLPFC rTMS treatment, increased [oxy-Hb] values in the bilateral frontopolar prefrontal cortex (FPPFC), ventrolateral prefrontal cortex (VLPFC) and left DLPFC during the VFT were observed in depressed patients. The increased [oxy-Hb] values from baseline to post-treatment in the right VLPFC in depressed patients were positively related to the reduction of HAMD score following rTMS.

**Conclusion:**

These findings suggest that the function of the prefrontal cortex in depressed patients was impaired and could be recovered by 2 Hz rTMS. The fNIRS-measured prefrontal activation during a cognitive task is a potential biomarker for monitoring depressed patients’ treatment response to rTMS.

## Introduction

Major depressive disorder (MDD) is a common psychiatric disease, with over 300 million individuals worldwide suffering from the disease (1). Unfortunately, as yet there is no specific biomarker for diagnosing and monitoring the progression of depression. Moreover, although pharmacotherapy is the first-line antidepressant treatment, about a third of patients with MDD are failed to achieve satisfied response to the initial antidepressant treatment because of the ineffectiveness or side effects of antidepressant medications (2).

For patients with MDD, repetitive transcranial magnetic stimulation (rTMS) has emerged as a promising treatment (3, 4). rTMS is a safe and non-invasive brain stimulation techniques for stimulating specific cortical regions and modulating neuronal activity (5). During the last decades, a great number of studies have demonstrated encouraging results about the utility of rTMS in neuropsychiatry (6–9). And the U.S. Food and Drug Administration has approved the rTMS applied over the dorsolateral prefrontal cortex (DLPFC) for MDD in 2008. However, the neural mechanism of rTMS treatment in depression is not very clear. The current application principle of rTMS is based on human neurophysiological experiments using motor evoked potential (MEP), i.e., high-frequencies rTMS (typically 5 or 10 Hz) demonstrates an excitatory effect on the stimulated motor cortex (M1), while low-frequencies rTMS (typically 0.2 to 1 Hz) suppresses cortical excitability of the stimulated M1 (10). The knowledge learned from stimulation of M1 are assumed to be applicable in other cortical regions, such as the prefrontal cortex (PFC), where the physiological response of stimulation is difficult to measure using MEP-related outcomes. Due to the lack of an objective marker, it is difficult to determine the optimal stimulation parameters in the PFC regions and evaluate the immediate and long-term responses to rTMS treatment.

Along with the rapid development of recent neuroimaging technologies, the brain activity in patients with MDD has gradually come to be visible. Previous functional neuroimaging studies have shown the dysfunction of the PFC in patients with MDD, which may be related to their clinical symptoms, including both depressive mood and cognitive impairment (11–13). Therefore, it is reasonable that the combined use of rTMS and neuroimaging will provide a reliable evaluation of neurobiological state, and their combination will facilitate understanding of potential modulation over the time course of rTMS treatment in a depressive brain. Functional near-infrared spectroscopy (fNIRS) is an emerging optical neuroimaging technology that can measure changes in concentrations of oxygenated hemoglobin [oxy-Hb] and deoxygenated hemoglobin [deoxy-Hb] in the brain cortex (14). Compared with positron emission tomography (PET) and functional magnetic resonance imaging (fMRI), fNIRS has several advantages in that it is easy to use and has a low cost. Accordingly, fNIRS has been widely used in clinical application and medical research to assess cortical functions of patients with psychiatric disorders.

The verbal fluency task (VFT) is a frequently used cognitive task in the fNIRS studies, in which participants are asked to generate as many words as possible beginning with a certain semantic category or letter within a limited time. The VFT mainly reflects the executive function, which is associated with some basic neurocognitive activities, such as working memory, motivation and attention. Many studies have reported that neurocognitive impairments are associated with PFC dysfunctions in numerous psychiatric disorders (15, 16). Accordingly, VFT has been widely employed in psychiatric disorders as a sensitive indicator of deficits in cognitive and executive domains that depend on the activation of prefrontal regions. A large number of fNIRS studies (17) reported that the [oxy-Hb] activation in the PFC during VFT was lower in MDD patients than in healthy controls, suggesting the executive dysfunction in patients with MDD may be caused by the impairment of the PFC functioning. Based on these findings, fNIRS is a promising technique for evaluating cortical functional changes in real time.

In past decades, two main rTMS strategies for depression treatment have been developed: high-frequency rTMS on the left DLPFC and low-frequency rTMS on the right DLPFC (6, 18, 19). Although both protocols have been shown be equally antidepressant effective as standard antidepressant medications (20–22), their therapeutic effects appear to be moderate. Therefore, an increasing number of studies have explored novel rTMS protocols for achieving better therapeutic efficacy. Among them, Fitzgerald et al. (23) reported that 2 Hz right DLPFC protocol was slightly superior to 1 Hz right DLPFC protocol in reducing the depressive symptoms in MDD patients. The finding led us to explore the antidepressant effect of 2 Hz rTMS over the right DLPFC and its possible neural mechanism.

In our study, fNIRS was used to examine the hemodynamic changes in the PFC in both patients with MDD and healthy counterparts during VFT, and then patients were treated with 4-week of 2 Hz right DLPFC rTMS treatment. We compared the change in the PFC before and after rTMS treatment. We hypothesized that (1) patients with MDD demonstrate a reduced activation of the PFC area during VFT, compared with their healthy counterparts, and (2) the level of activation in the PFC and the severity of depressive symptoms can be improved by using 2 Hz DLPFC rTMS treatment.

## Materials and Methods

### Participants

Patients were recruited from the outpatient department of West China Hospital, Sichuan University from May 2021 to December 2021. We included 40 patients aged 20–59 years who were diagnosed with moderate MDD according to the fifth edition of the Diagnostic and Statistical Manual of Mental Disorder (DSM-5) with a Hamilton Rating Scale for Depression-24 item (HAMD-24) total score between 20 and 35. All patients had to be right-hand dominant. In order to eliminate the influence of antidepressant medications, we included the patients who had not taken antidepressants at least 1 month before treatment start. Considering that it is common for patients with depression comorbid with anxiety and insomnia (24, 25), the use of benzodiazepines was allowed in this experiment since it can alleviate anxiety and insomnia but not depression. The exclusion criteria were as follows: (i) severe and unstable physical illnesses; (ii) antidepressants have been used within 4 weeks before enrollment; (iii) had a score ≥3 on item 3 (suicidal thoughts) of the HAMD-24 or had made a suicide attempt in the previous 6 months; (iv) presence of other mental disorders; (v) severe auditory dysfunction; (vi) pregnant or breastfeeding women; and (vii) contraindications for undergoing rTMS treatment, such as metallic implants or a history of epileptic seizure.

Forty healthy controls (HCs) were recruited from the local community and matched to the MDD patients in terms of age, gender, level of education. They were required to be right-hand dominant and in a good healthy condition with no any known history of neurologic and psychiatric diseases, or a family history of psychotic disorder.

Before this study, all participants provided written informed consent. This study was approved by the West China Hospital Clinical Trials and Biomedical Ethics Committee of Sichuan University (No. of ethical approval: [2021]-428) on April 30, 2021 and registered in the Chinese Clinical Trials Registry (registration No. ChiCTR2100046806) on May 29, 2021.

### Study Overview

Major depressive disorder patients received rTMS treatment for 4 weeks. Depression severity was assessed by HAMD-24. Before and after 4 weeks of rTMS treatment, all patients were assessed with HAMD-24 and fNIRS by a trained research staff to ensure consistency. In addition, considering acute cognitive enhancing effects of rTMS, which were typically observed when the test was administered immediately following stimulation, usually within several minutes (26), we thus set the interval between the last rTMS session and fNIRS test after treatment more than 24 h in this study. Healthy controls were only assessed with fNIRS at baseline.

### Repetitive Transcranial Magnetic Stimulation Intervention

Repetitive transcranial magnetic stimulation was performed with a CCY-I Magnetic Stimulator (YIRUIDE Medical Co., Wuhan, China) with an air-cooled, figure-of-eight 70 mm coil. At the first TMS session, the resting motor threshold (RMT) of the right abductor pollicis brevis muscle was determined as the lowest strength of transcranial magnetic stimulation needed to elicit at least 5 electromyographic responses in the form of motor evoked potential (EMG/EP Measuring system, Nihon Kohden, Tokyo, Japan) ≥50 μV in 10 trials (27). The site of stimulation during the TMS treatment sessions was right DLPFC defined by a point 5 cm anterior to the motor hotspot (28). Treatment parameters were standardized for each session at the treatment location with the following stimulation parameters: 90% of individual RMT, frequency in 2 Hz, train duration of 10 s, inter-train interval of 3 s and 130 trains per session, leading to a total of 2,600 pulses delivered in 28.7 min. The treatment was performed 5 days per week for 4 weeks for a total of 20 sessions.

### Clinical Assessment

The 24-item HAMD (29, 30) includes 24 items rated on either a 2-, 3- or 4- point scale with total score range from 0 to 76 points. Patients who achieve a HMAD-24 total score of 8–19 points are regarded as mild depression, total score of 20–35 points are regarded as moderate depression, total score of >35 points are regarded as severe depression (31). As a note, HAMD was used to evaluate the level of depression, but rather to offer a strict diagnostic guideline.

The primary outcome measure for this study was the total score of HAMD-24. Clinical response was defined as a reduction in HAMD-24 scores of at least 50% from baseline.

### Activation Task (Verbal Fluency Task)

**FIGURE 1:**
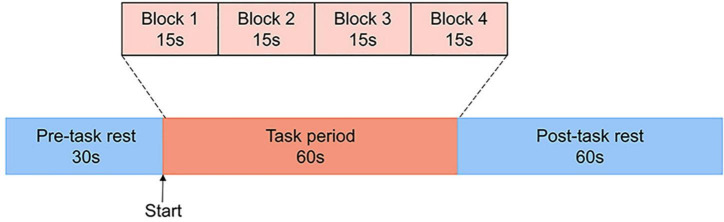
The VFT protocol used for near-infrared spectroscopy. Each trial consisted of a 30s pre-task rest period, a 60s task period subdivided into four 15s blocks and finally, a 60s post-task rest period.

The task procedure in the present study was a Chinese-language phonological VFT developed by Quan et al. (32) for Chinese participants. Previous research (33) has shown evidence that patients with MDD are associated with reduced brain activation in the prefrontal cortex during this version of VFT in comparison to the healthy controls. Each trial consisted of a 30s pre-task rest period, a 60s task period and finally, a 60s post-task rest period (see Figure 1). During the pre-task and the post-task rest period, participants were asked to verbally count the numbers from one following the voice prompts from the fNIRS machine. The 60s task period was divided into four sequential 15s blocks. During each 15s block, one of four Chinese syllables “shang (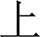),” “shi (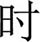),” “shuo (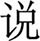),” and “jia (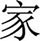),” which indicate upper, time, speak and home, respectively, was audibly presented to the subjects. And subjects were instructed to generate as many words as possible which began with the same syllable. All the participants were given the same syllable cues and no changes were made to the order of presentation. We provided all participants with a practice session before the formal testing, in order to ensure the participants fully understand the tasks. During the task, an investigator monitored the performance of the participants, in order to ensure the participants were fully engaged in the assessment.

### NIRS Measurement

The 37 multi-channel fNIRS instrument (BS-3000, Wuhan Union Medical Technology Co., Wuhan, China) measures the concentration changes of [oxy-Hb] and [deoxy-Hb] in cerebral cortex using two wavelengths (695 and 830 nm) of infrared light, based on the modified Beer Lambert law (34). The absorption of those infrared light emitted by dual wavelength laser diodes could distinguish the deoxyhemoglobin and oxyhemoglobin (35). This system consists of 12 light emitters and 12 light detectors, and the distance between each emitter and detector is 3 cm. A channel (ch) was defined as the measurement area between a detector and source probe pair. The sampling rate was set to 20 Hz. The probe set was positioned on the participants’ prefrontal areas and the lowest probes were positioned along the Fp1–Fp2 line in accordance with the International 10–20 System of electroencephalogram electrode placement. Thus, the waveforms change of [oxy-Hb] and [deoxy-Hb] in PFC were acquired from all 37 channels.

**FIGURE 2:**
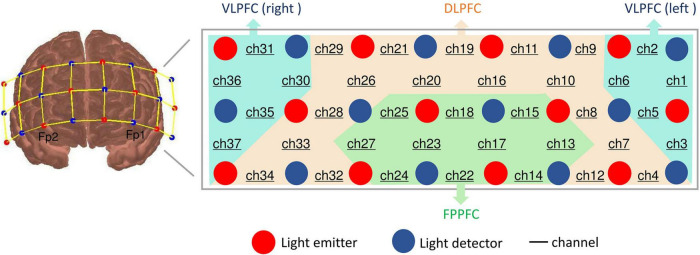
Location of probes and channel settings in 37-channel near-infrared spectroscopy. Brodmann area showing 37 sensing areas (from Ch1 to Ch37) within the prefrontal cortex.

According to a previous study of anatomical craniocerebral correction via the international 10–20 system (36, 37), we confirmed the correspondence between the NIRS channel and the measurement position on the cerebral cortex. Thus, according to the international 10–20 system, the approximately positions of the 37 channels were as follows: ch4, 7–12, 16,19–21, 26, 28, 29, and 32–34 are located over the DLPFC (BA 9 and 46), ch1-3, 5, 6, 30, 31, and 35–37 are located over the ventrolateral PFC (VLPFC; BA 44, 45, and 47) and ch13-15, 17, 18, 22–25, and 27 are located over the frontopolar PFC (FPPFC; BA 10), based on Brodmann’s area (BA) (38) (see Figure 2).

### NIRS Data Analysis

The toolbox HOMER2, a MATLAB-based graphical user interface program was used to analyze the NIRS data (39). First, the raw data were filtered using a Band-pass filter within the range of 0–0.1 Hz to remove high frequency noise. Similar to study of Lee et al. (40), the threshold signal-to-noise ratio in our study was 30 dB, which was used to qualify the noise of the detected channels after band-pass filtering and eliminate the slow drift of physiological and environmental noise. Then, a processing method based on moving standard deviation and cubic spline interpolation was applied to remove motion artifacts (41, 42). Artifacts were distinguished by identifying the sliding window standard deviation above a certain threshold and were removed by cubic spline interpolation (43). Finally, the filtered optical data were translated to [oxy-Hb] concentrations by applying the modified Beer-Lambert law (44). We focused on [oxy-Hb], since the change of [oxy-Hb] could better reflect cortical activity as it is assumed to more directly response to cognitive task-related brain activation and more strongly correlated with blood oxygenation level dependent (BOLD) signals measured by fMRI (45). And we took the final 10 s of the pre-task rest period as the baseline. The mean [oxy-Hb] values of the task period and baseline in each channel for each participant were calculated separately. The [oxy-Hb] values during the VFT, i.e., the mean [oxy-Hb] change between the baseline and task period., was finally calculated by subtracting the baseline mean [oxy-Hb] values from the task period mean values.

### Statistics

Statistical analysis was performed using SPSS software version 26.0. Baseline demographic characteristics were assessed by means of a chi-squared test (sex), *t*-test (age) or Mann-Whitney test (education), comparing the MDD and HCs groups. Symptom change, i.e., difference between the two total HAMD-24 scores (pre – post over the full treatment course), was compared by paired *t*-test. To analyze our NIRS data, Mann-Whitney *U* tests were used to compare [oxy-Hb] values during the VFT for each channel between the MDD and HCs groups at baseline. Then, to assess [oxy-Hb] responses to rTMS treatment in patients with depression, the differences in [oxy-Hb] values during the VFT for each channel were compared between pre- and post-treatment in the MDD group, using Wilcoxon test.

To examine the relationships between [oxy-Hb] values before treatment during the VFT and HAMD-24 total scores and to test whether the former was related to clinical outcome, correlation analysis was carried for MDD patients. For channels showing a significant difference in [oxy-Hb] value in MDD group between pre- and post-treatment, we also examined the association between the [oxy-Hb] changes from baseline to post-treatment and the degree of improvement in depressive symptoms.

We adopted a false discovery rate (FDR) (46) in order to perform multiple comparisons for the neural activation in the probes of 37 channels. Significance level was set at a *p* < 0.05.

## Results

### Demographic and Clinical Characteristics

There was no significant difference in gender (chi-square test: χ^2^ = 0.621, *p* = 0.600), age (*t*-test: *t* = 0.247, *p* = 0.806) or education (Mann-Whitney test: *z* = −0.036, *p* = 0.971) between the MDD patients and the healthy controls. For patients, the duration of illness was 5.80 ± 7.37 years. A 60% (24/40) patients were diagnosed with MDD with a first episode and 40% (16/40) had recurrent episodes. A 72.5% (29/40) patients were comorbid with anxiety and 75% (30/40) patients were comorbid with insomnia. A 67.5% (27/40) patients had never taken antidepressant medications. A 55% (22/40) patients were medicated with benzodiazepine drugs. The demographic and clinical characteristics of the participants are presented in Table 1.

**TABLE 1:** Demographic and clinical characteristics of participants.

	HCs	Patients with MDD		*t/z/χ^2^*	*p*
		Pre-treatment	Post-treatment		
**n**	40	40	40	–	–
**Demographic**					
Age, years	37.75 ± 4.72	38.18 ± 9.81	–	0.247	0.806
Gender, male/female, n	11/29	8/32	–	0.621	0.600
Education, years	13.93 ± 2.00	13.83 ± 3.01	–	−0.036	0.971
**Clinical**					
Duration of disease, years	–	5.80 ± 7.37	–	–	–
**Number of episodes, n (%)**					
Single episode		24 (60%)			
Recurrent episode		16 (40%)			
**Comorbidity, n (%)**					
Anxiety		29 (72.5%)			
insomnia		30 (75%)			
Previous antidepressants history, yes/no		13/27			
Current benzodiazepine use, yes/no		22/18			
HAMD-24 scores	–	26.08 ± 4.66	17.73 ± 8.12	6.700	0.000

### Clinical Outcomes

After 4-weeks of rTMS treatment, the HAMD-24 scores in the MDD patients significantly decreased, from 26.08 to 17.73 (paired *t*-test: *p* < 0.001). A 27.50% (11/40) of MDD patient were responded to treatment in our study.

### Effects of Repetitive Transcranial Magnetic Stimulation on [oxy-Hb] Signals During the Verbal Fluency Task

**FIGURE 3:**
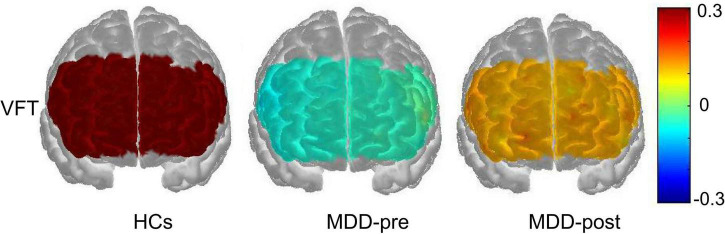
Prefrontal cortical activation during the VFT in patients with MDD before (pre) and after (post) rTMS treatment and HCs. The color scale depicts the change of [oxy-Hb] value range from −0.3 to 0.3 in μmol × mm.

**FIGURE 4:**
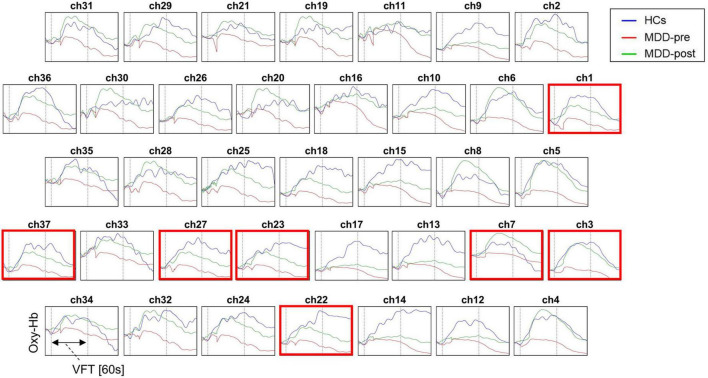
Waveforms of [oxy-Hb] values during the VFT in the 37 channels over prefrontal regions in patients with MDD before (pre) and after (post) rTMS treatment and HCs. Red box indicates significant increase in [oxy-Hb] value from baseline to post-treatment in patients with MDD in this channel.

At baseline, the [oxy-Hb] values in the MDD group during the VFT were significantly lower than that of HCs group in the 31 channels located over the bilateral FPPFC, DLPFC and VLPFC (HCs vs. MDD-pre: ch1-18, 20–28, 32–34 and 37; Mann-Whitney test: *z* = −5.965 – −2.212, FDR *p* = 0.00001–0.032). After 4-weeks of rTMS treatment, the significant increase in [oxy-Hb] changes were observed in the MDD group compared with the pre-treatment levels in the 7 channels located over the bilateral FPPFC, left DLPFC and bilateral VLPFC (MDD-pre vs. MDD-post: Ch1, 3, 7, 22, 23, 27, and 37; Mann-Whitney test: *z* = −3.669 – −2.594, FDR *p* = 0.0002–0.047). The prefrontal cortical activation during the VFT in different group are shown in Figure 3. Figure 4 shows the waveforms of [oxy-Hb] values during the VFT in 37 channels over prefrontal regions in different group.

### Correlation Between NIRS Data and Clinical Data

**FIGURE 5:**
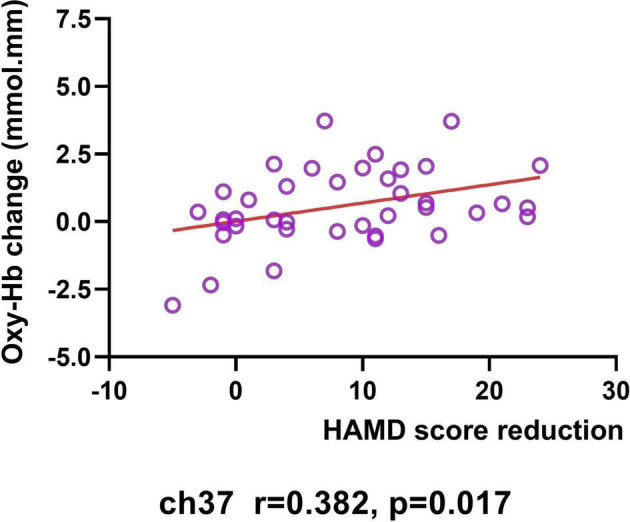
Correlation between HAMD score reduction and changes in [oxy-Hb] value (from baseline to post-treatment) in Ch37.

In the MDD group, the increased [oxy-Hb] values from baseline to post-treatment was positively related to the total HAMD-24 score reductions in the right VLPFC in 37 (Pearson’s *r* = 0.381, *p* = 0.017; see Figure 5). However, the [oxy-Hb] values before treatment were not significantly correlated with baseline HAMD scores, nor the total HAMD-24 score reductions after treatment (all *p* < 0.05).

However, after FDR correction, there was no significant correlations between NIRS data and clinical data.

## Discussion

To the best of our knowledge, this is the first study to examine the effects of DLPFC rTMS on modulating to cognitive task in MDD patients using fNIRS. We found that patients with MDD had significantly less activation in the PFC in response to the VFT compared to their healthy counterparts, whereas that the level of the activation in patients was not related to their depression severity. After 4-weeks of 2 Hz DLPFC rTMS treatment, the decreased depressive severity and the increased activation in response to the VFT were observed in MDD patients, and the increased activation in the right VLPFC from baseline to post-treatment was positively related to the improvement of depressive symptoms.

The overall results of previous NIRS studies of the VFT revealed reduced PFC activation in MDD patients compared with HCs (33, 40, 47, 48). Akiyama et al. (49) demonstrated that the hemodynamic response to the VFT in MDD patients was significantly reduced compared with HCs in the bilateral DLPFC (BA 9, 46), VLPFC (BA 44, 45, 47), and FPPFC (BA 10) cortical surface regions. Consistent with those previous studies, our study also found less activation in the different PFC areas (the bilateral FPPFC, DLPFC and VLPFC) in MDD patients during the VFT was, suggesting the functional hypofrontality in the bilateral PFC in patients with MDD. Similarly, several fMRI studies show that the MDD patients had a reduced response in the PFC, particularly in the left DLPFC, during the VFT compared to the healthy controls (50, 51). The findings may be attributed to neuronal dysfunction through the mechanism of neurovascular coupling (52), or a decreased cerebral vasoreactivity (53). In accordance with the study of Tsujii et al. (54), our study observed no significant correlations between HAMD scores and oxy-Hb concentrations. However, the results about the clinical correlations between [oxy-Hb] change on NIRS and depression symptom severity were still controversial as several studies (55–57) suggested that a correlation existed, while others did not. The difference between these results may be related to the inconsistency in the patient characteristics between studies and differences in the methods used to analyze the [oxy-Hb] variations measured by fNIRS.

We found that the [oxy-Hb] values in the bilateral FPPFC, left DLPFC and bilateral VLPFC during the VFT were gradually increased after 4-weeks of 2 Hz rTMS applied on right DLPFC than before rTMS. Our results suggest that 2 Hz rTMS could evoke an increased cerebral cortex activation during a cognitive task. Similarly, previous study using fMRI indicated that 2-week of rTMS applied on the DLPFC had focal and remote effects on several brain areas involved in working memory in healthy subjects during an n-back task (58). Also, Cao et al. (59) investigated the effect of 5s trains of 1, 2, and 5 Hz stimulation delivered at the left DLPFC on twelve healthy participants, showing a decrease in blood oxygenation after 1 Hz compared to the [oxy-Hb] increases observed in both the 2 and 5 Hz stimulations. Although several NIRS studies (60, 61) have also investigated the effects of rTMS on the MDD patients and described a modulation of the blood oxygenation response over the PFC that was built up during the course of rTMS treatment in depression, these studies measured the oxy-Hb response during TMS, not during a cognitive task. Given the interest in using rTMS to influence high-level cognitive function, the changes in functional measures during task-related activity are particularly important. Moreover, the present study differed from previous studies in that the patients we included were not taking antidepressant. In addition to the uncertain effects on cognition, studies have found that antidepressant can affect the NIRS signals (62). Thus, a strength of our study is that fNIRS data we collected was not interfered by medicine.

In the present study, 2 Hz right DLPFC rTMS was effective as a monotherapy for MDD who were not undergoing any antidepressant medication. The finding was consistent with previous studies (63, 64), which demonstrated the improvement of depressive symptoms in patients with treatment-resistant depression after 2–4 weeks of rTMS treatment. In the study of Fitzgerald et al. (23), 42% patients in the 1-Hz group and 53% patients in the 2-Hz group achieved response criteria. A meta-analysis reported response rates of 45% (144/320) in patients treated with low frequency right-sided TMS and 48% (148/307) in patients treated with high frequency left-sided TMS (65). The lower response rate in our study was 27.5% which was relatively lower than the findings of previous studies. It may be because the patients in our study were undergone monotherapy treatment of rTMS without medications, or the differences in the characteristics of our sample (i.e., moderate depression) and stimulation protocol applied.

Moreover, our study demonstrated, for the first time, the correlation of the increase of NIRS activation in the prefrontal region with improvements in the depressive symptoms of patients during the rTMS treatment. In our 4-week rTMS treatment period, the longitudinal increases in the right VLPFC were shown to be positively correlated with improvements in the severity of depressive symptoms for MDD patients. This finding is consistent with the previous study conducted by Shinba et al. (61) which investigated the relationship between cerebral blood flow changes during stimulation and the effectiveness of TMS. Their result showed that increased PFC oxy-Hb levels during TMS at the last day of treatment were linked to a larger reduction of depressive symptoms. As such, they also concluded that the maintenance of PFC activation during stimulation in the course of TMS series is related to the effectiveness in the treatment of depression. Although the significant result in our study did not survive FDR correction for multiple, our discovery partly suggested that fNIRS could be useful in monitoring treatment response of rTMS treatment in patients with MDD. Also, to some extent, our observations support the potential neuroimaging mechanism of DLPFC-rTMS treatment in MDD, namely increased metabolic activity and blood flow perfusion in frontal regions (66, 67).

## Limitations

There are some limitations of our study. First, our fNIRS signals in typical source-detector channels were possibly contaminated with systemic interference occurring in the superficial layers of the head (68). Although the brain hemodynamics response to a task without short channel separation has been used in depression assessment (69), it can be more precise to use an additional short source-detector separation optode in future study, to order to remove the systemic interference and improve the accuracy of fNIRS measurements (70). Second, the duration of follow-up in the present study was not long enough; Having more frequent fNIRS measurements in longitudinal studies (e.g., weekly) may oxygen hemodynamics provide us with a better understanding of the brain dynamics and minimize the influence of confounding factors. Third, longer longitudinal studies of at least 6 months to 1 year would be beneficial, considering that most depressive episodes last for at least a few months. Fourth, most of patients in this study comorbid with anxiety and dysthymia, which could affect the NIRS assessment results. Fifth, our sample size was small. Sixth, we did not employ a sham rTMS-control group. Considering these limitations, future studies with larger sample sizes and placebo-control participants are needed to confirm our preliminary findings for 2 Hz rTMS, especially with respect to an intensified PFC hemodynamic response.

## Conclusion

Our study demonstrated that patients with MDD had significantly reduced brain activation in the PFC during VFT when compared with HCs, and these functional deficits can be improved after 4 weeks of 2 Hz right DLPFC rTMS treatment. Furthermore, there was correlation between improvements in the depression severity in MDD and increases of hemodynamic response to VFT in the right VLPFC during treatment. These results suggest that hemodynamic response to VFT in PFC, measured by fNIRS, is a potential biomarker for monitoring MDD patients’ treatment response to rTMS. How to improve the cognitive and brain function of MDD and how to predict the prognosis of the patients are important issues that need more exploration in the future.

## Data Availability Statement

The original contributions presented in the study are included in the article/supplementary material, further inquiries can be directed to the corresponding authors.

## Ethics Statement

The studies involving human participants were reviewed and approved by the West China Hospital Clinical Trials and Biomedical Ethics Committee of Sichuan University. The patients/participants provided their written informed consent to participate in this study.

## Author Contributions

ZZ conceived and designed the experiments. JH performed the experiments and data analysis. TZ checked the processed experimental data. JZ and JH wrote the manuscript with input from all authors. PW supervised the project. All authors discussed the results and contributed to the final manuscript.

## Conflict of Interest

The authors declare that the research was conducted in the absence of any commercial or financial relationships that could be construed as a potential conflict of interest.

## Publisher’s Note

All claims expressed in this article are solely those of the authors and do not necessarily represent those of their affiliated organizations, or those of the publisher, the editors and the reviewers. Any product that may be evaluated in this article, or claim that may be made by its manufacturer, is not guaranteed or endorsed by the publisher.
